# Somatic infraciliature in tintinnid ciliates (Alveolata, Ciliophora, Spirotricha): An ultrastructural comparison*

**DOI:** 10.1111/jeu.12885

**Published:** 2022-01-30

**Authors:** Sabine Agatha, Michael S. Gruber, Heidi Bartel, Birgit Weißenbacher

**Affiliations:** ^1^ Department of Environment & Biodiversity Paris Lodron University of Salzburg Salzburg Austria; ^2^ Hieronymus‐Illustrations Salzburg Austria

**Keywords:** Apomorphy, choreotrichids, Oligotrichea, phylogeny, somatic kinetids

## Abstract

A recent ultrastructural study on the tintinnid ciliate *Schmidingerella meunieri* displayed unique types of somatic kinetids. The dikinetids (paired basal bodies) have, besides kinetodesmal fibrils and transverse ribbons, some special features, that is, overlapping postciliary ribbons and three extraordinary microtubular ribbons, which together form a conspicuous network in the ciliated anterior cell portion. The distribution of this feature among tintinnids is studied in chemically fixed and ultrathin‐sectioned specimens from six genera and five families collected in European coastal waters. The taxa are scattered across the molecular tree. Actually, the somatic kinetids of these six genera share the special features discovered in *S*. *meunieri*. Accordingly, the overlapping postciliary ribbons and the three extraordinary ribbons were already present in the early stages of tintinnid evolution, namely in the last common ancestor of tintinnids with hard loricae. Owing to the lack of ultrastructural data in the basally branching Tintinnidiidae with their soft loricae and in aloricate choreotrichids other than the aberrant strobilidiids, the first appearance of the structures is still uncertain. The related oligotrichids do not possess overlapping postciliary ribbons, but show electron‐dense material at the sites where the ribbons I–III originate in tintinnids. None of these features is found in any other spirotrich ciliate.

The ultrastructure of somatic kinetids is generally considered a rather conservative feature in ciliates allowing the characterisation of higher systematic ranks (Lynn, 1981; Lynn & Small, 2000; Small & Lynn, 1981, 1985); molecular phylogenies actually confirmed most of these taxa (Lynn, 2017). According to Adl et al. (2019), the spirotrich ciliates comprise the licnophorids, kiitrichids, euplotids, hypotrichs, oligotrichids, and choreotrichids; the latter three taxa are united in the Perilemmaphora based on the shared possession of a perilemma, an additional membranous structure embracing the cell. The oligotrichids and choreotrichids are adelphotaxa, and the latter contain the monophyletic lorica‐forming tintinnids besides aloricate forms; the phylogenetic placement of the enigmatic species *Lynnella semiglobulosa* is currently uncertain. The sparse ultrastructural data on spirotrichs, however, contradict the structural conservatism of the somatic kinetids (Table 1; Lynn, 2008, 2017). The recent findings on the tintinnid *Schmidingerella meunieri* even enhanced the previously reported diversity: the somatic kinetids of this ciliate display besides the plesiomorphic features (kinetodesmal fibril, postciliary ribbon, and transverse ribbon) surprising apomorphies, that is, overlapping postciliary ribbons and three extraordinary microtubular ribbons (Gruber et al., 2018). These microtubular ribbons generate jointly a conspicuous network in the ciliated anterior cell portion of the tintinnid. The present comparative approach on six further genera and four additional families aims to ascertain whether the network and its constituting ribbons are autapomorphies of the genus *Schmidingerella* or have a wider distribution among tintinnid ciliates. Independent of the nuclear marker gene analysed (18S rDNA, ITS, and 28S rDNA), the Tintinnidiidae, Tintinnidae, Eutintinnidae, and Favellidae consecutively branch off in the phylogenetic tree, while the remaining tintinnids form several statistically well‐supported clades whose relationships are currently uncertain (Santoferrara & McManus, 2021).

**TABLE 1:** Ultrastructural comparison of dikinetids (paired basal bodies plus associated structures) and monokinetids (single basal bodies plus associated structures) forming ciliary rows in spirotrich ciliates

Taxon	Kinetid structure	References
Licnophorids	Dikinetids: anterior basal body with two transverse ribbons and a spur of electron‐dense material, posterior basal body with two ‘postciliary’ ribbons, transverse ribbon, and kinetodesmal fibril	da Silva Neto (1994)
Kiitrichids	Dikinetids: anterior basal body with transverse ribbon, posterior basal body with postciliary ribbon and kinetodesmal fibril	Fleury et al. (1985b, 1986)
Euplotids	Dikinetids: anterior basal body with transverse ribbon, posterior basal body with postciliary ribbon and kinetodesmal fibril	Görtz (1982), Lenzi and Rosati (1993), Morelli et al. (1996), Wicklow (1983)
Hypotrichs	Dikinetids: anterior basal body with transverse ribbon, posterior basal body with postciliary ribbon and transient kinetodesmal fibril	Fleury et al. (1985a), Grimes and Adler (1976)
Oligotrichid *Limnostrombidium viride*	Dikinetids: anterior basal body with electron‐dense material (extraordinary ribbon III?), posterior basal body with postciliary ribbon, kinetodesmal fibril, and electron‐dense material (extraordinary ribbons I and II?)	Bardele et al. (2018)
Aloricate choreotrichid *Rimostrombidium lacustre*	Monokinetids with double‐rowed ‘transverse’ ribbon	Grim (1987; reported as *Strobilidium velox*)
Tintinnid *Schmidingerella meunieri*	Dikinetids: anterior basal body with transverse ribbon and extraordinary ribbon III, posterior basal body with overlapping postciliary ribbon, kinetodesmal fibril, and extraordinary ribbons I and II Monokinetids with overlapping postciliary ribbon, kinetodesmal fibril, and extraordinary ribbons I and II	Gruber et al. (2018)

## MATERIALS AND METHODS

*Amphorides minor* (Tintinnidae), *Cyttarocylis* sp. (Cyttarocylididae), *Dadayiella ganymedes* (incertae sedis in Xystonellidae), *Eutintinnus elongatus* (Eutintinnidae), and *Rhabdonella spiralis* (Rhabdonellidae) were collected from the plankton of the bight of Villefranche‐sur‐Mer (43°42′N, 07°19′O), Côte d’Azur, France, in October 2016 at a salinity of about 40‰ and a water temperature of about 21°C (Figure S1B–D,F). *Helicostomella subulata* (incertae sedis in Tintinnina; tintinnid clade 11 according to Santoferrara et al., 2017) was sampled in Warnemünde (54°11′N, 12°05′O), Germany, at the Baltic Sea coast in October 2017 at a salinity of about 14‰ and a water temperature of about 14°C (Figure S1E). For details concerning species identifications, fixations and preparations for transmission electron microscopy, the reader is referred to Agatha and Bartel (2021) and for information regarding *Schmidingerella meunieri* (Figure S1A) to Gruber et al. (2018).

Briefly, the specimens were chemically fixed in 3% glutaraldehyde and 2% osmium tetroxide in 0.05 M cacodylate buffer in artificial seawater at a ratio of 1:1. The fixation was performed at the sampling sites owing to logistic reasons (the frequent death of the delicate specimens during the transport to the laboratory in Salzburg), except for *Schmidingerella meunieri*, in which culture material allowed a cryofixation providing excellent results (Gruber et al., 2018). The polymerised samples were ultrathin‐sectioned (70 nm) with an ultramicrotome (Leica EM UC7). The investigations were conducted by means of a Zeiss EM 910 transmission electron microscope (Karl Zeiss AG). The micrographs were taken with a Sharp:Eye digital camera system (Tröndle), using the computer software ImageSP Viewer (SysProg & TRS). The database on the somatic kinetids in the taxa studied here comprises about 1970 images from 24 fixed specimens besides those of *S*. *meunieri* analysed by Gruber et al. (2018). The orientation of the micrographs is parallel to the cell's main axis, if not stated otherwise. All micrographs show the cell in surface view, and the terms ‘right’ and ‘left’ refer to the perspective of the cell, the organelle or the structure described. The terminology follows Gruber et al. (2018). Based on the findings in *S*. *meunieri*, a uniform ultrastructure of the dikinetids and monokinetids is also postulated for the tintinnids investigated here, although not all kineties could be studied. For measuring the inclination of the dikinetids in a kinety, a virtual line is drawn through the centres of the posterior basal bodies of two consecutive kinetids; the measurements are rough estimates and highly variable in all species possibly due to the curvature of the ciliary rows.

## RESULTS

For the first time, the ultrastructure of the somatic kinetids is presented in the tintinnid genera *Amphorides*,*Cyttarocylis*,*Dadayiella*,*Eutintinnus*,*Helicostomella*, and *Rhabdonella* (Table 2). To complete the present comparison, the findings on cryofixed and ultrathin‐sectioned cells of *Schmidingerella meunieri* published by Gruber et al. (2018) are briefly reported.

**TABLE 2:** Comparison of fibrillar associates of the somatic kinetids in seven tintinnid ciliates

Characters	*Schmidingerella*	*Amphorides*	*Eutintinnus*	*Dadayiella*	*Helicostomella*	*Cyttarocylis*	*Rhabdonella*
Dikinetids							
Kinetodesmal fibrils	+	+^a^	+	+	+	+	+
Overlapping postciliary ribbons	+	+	+	+	+	+	+
Transverse ribbons	+	+	?	?	?	?	+
Desmoses	+	+	+	+	+	+	+
Ribbons I‐III	+	+	+^a^	+	+	+^a^	+
Monokinetids							
Kinetodesmal fibrils	+	No monokinetids	+	+	+	+	+
Overlapping postciliary ribbons	+		+	+	+	+	+
Ribbons I + II	+		+	+	+	+	+

Note

The structures in *Schmidingerella meunieri* have been described in great detail by Gruber et al. (2018).

? Not or not clearly visible.

Present, but not or only partially shown in the micrographs of this publication.

**FIGURE 1:**
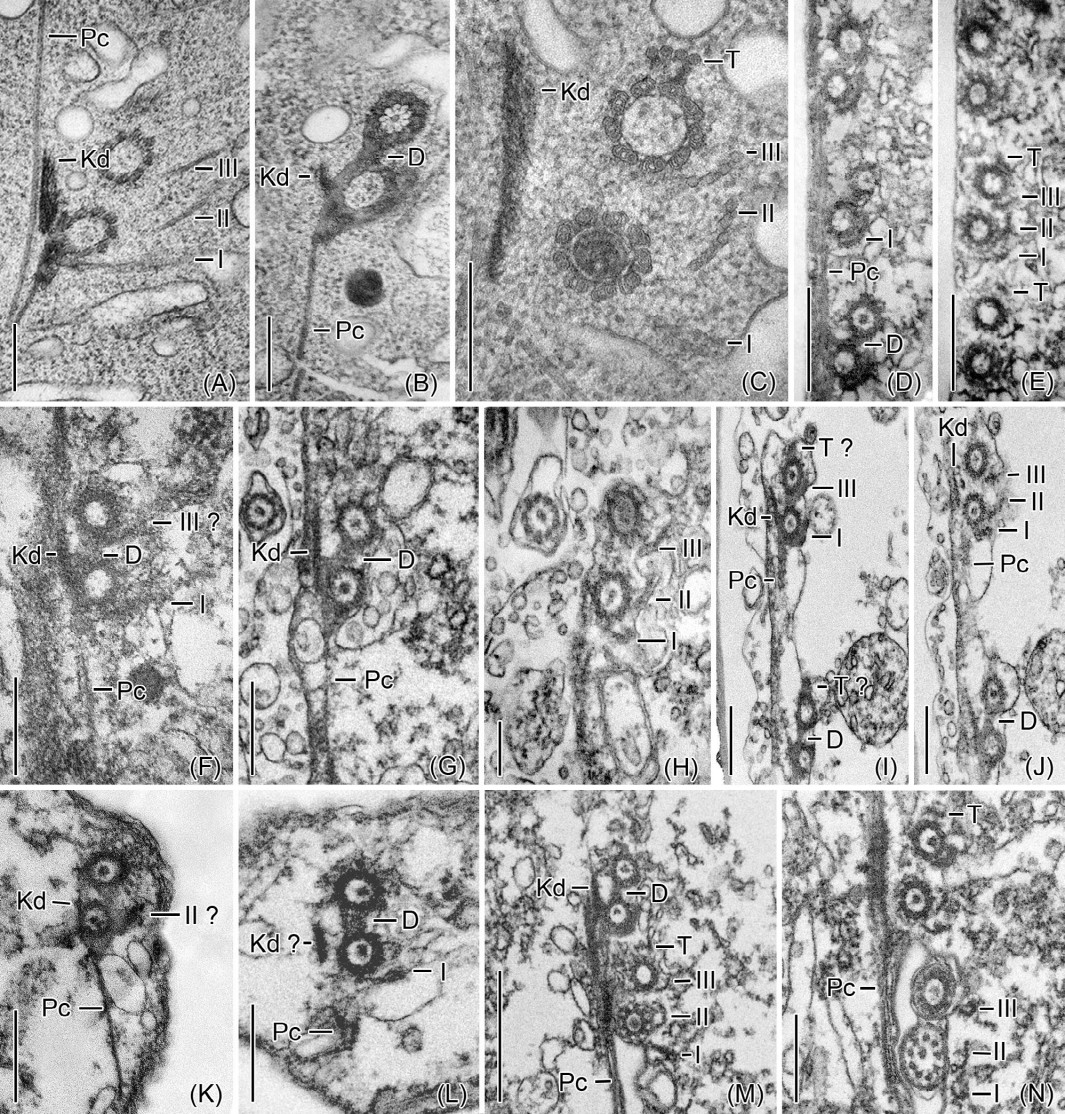
Transmission electron micrographs of dikinetids. The transverse sections at different levels show the same fibrillar associates in all species, except for the transverse ribbons, which are visible only in *Schmidingerella*,*Amphorides*, and *Rhabdonella*, while their presence is uncertain in the remaining taxa studied. (A–C) *Schmidingerella meunieri* [from Gruber et al. (2018)]. (D and E) *Amphorides minor*. (F) *Eutintinnus elongatus*. (G and H) *Dadayiella ganymedes*. (I and J) *Helicostomella subulata*. (K and L) *Cyttarocylis* sp. (M and N) *Rhabdonella spiralis*. I–III, extraordinary microtubular ribbons I–III; D, desmoses; Kd, kinetodesmal fibrils; Pc, postciliary ribbons; T, transverse ribbons. Scale bars = 250 nm (A–C, H), 500 nm (D–G, I–L, N), and 1 µm (M)

*Schmidingerella meunieri* (Kofoid and Campbell, 1929) Agatha and Strüder‐Kypke, 2012; Rhabdonellidae Kofoid and Campbell, 1929 (Table 2; Figures 1A–C, 2A,B, and 3A). The dikinetids are clockwise inclined, forming angles of 13°–41° (26° ± 9°; *n* = 11) to the kinety axes (Figure 1A–C). The posterior basal bodies have associated kinetodesmal fibrils, overlapping postciliary ribbons and two extraordinary ribbons on their left sides: the connective ribbons (ribbons I) and the posterior oblique ribbons (ribbons II). Their fibrillar associates are identical to those of the monokinetids (Figure 2A,B). The anterior basal bodies have associated transverse ribbons plus the extraordinary anterior oblique ribbons (ribbons III) on the left side. Desmoses link both dikinetidal basal bodies. Especially, the numerous ribbons I and II form together with the long overlapping postciliary ribbons a conspicuous subpellicular network in the ciliated anterior cell portion, in which the ribbons I abut on the next kinetids to the left (Figure 3A).

**FIGURE 2:**
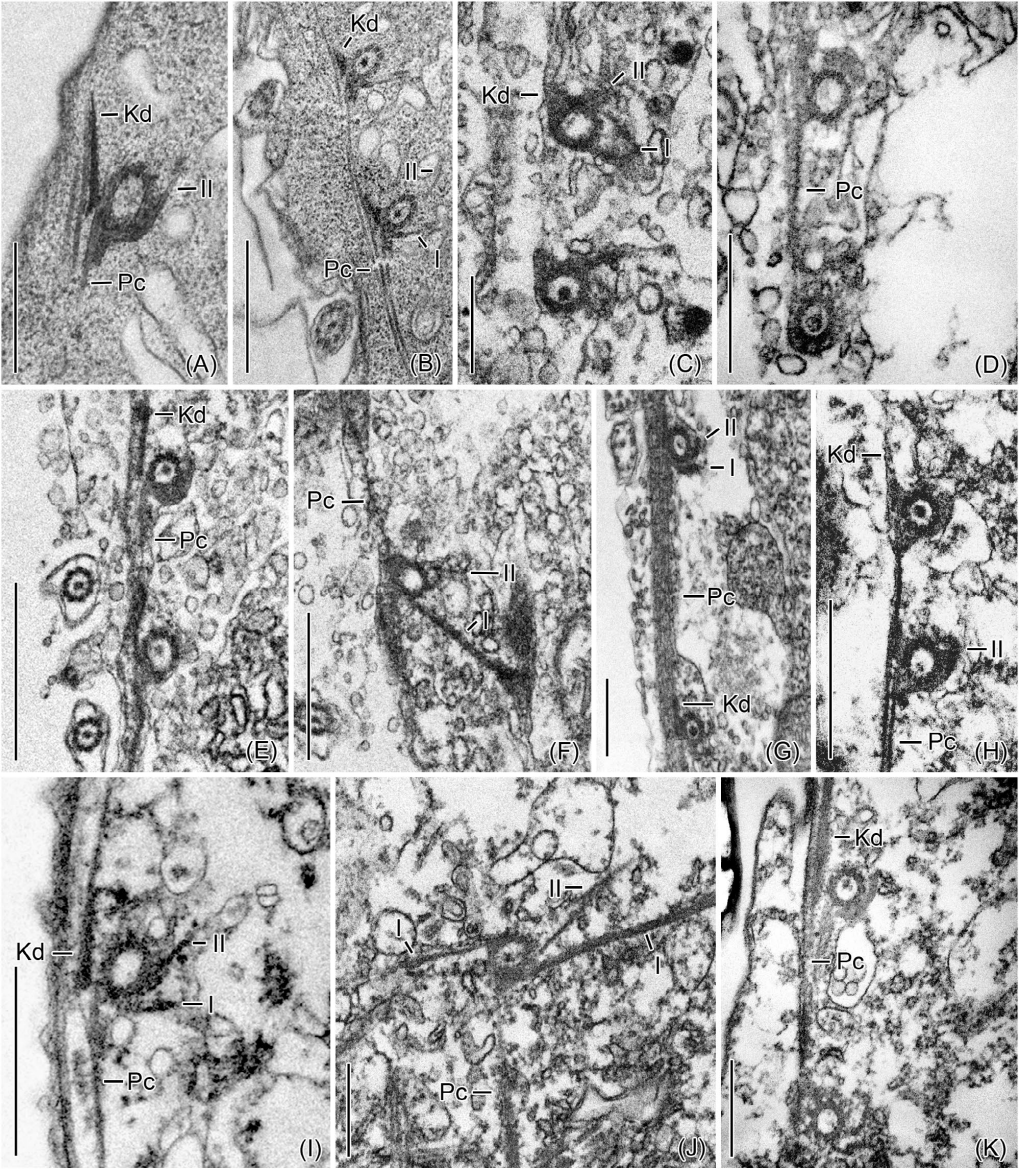
Transmission electron micrographs of monokinetids. The transverse sections at different levels show the same fibrillar associates in all species. (A and B) *Schmidingerella meunieri* [B, from Gruber et al. (2018)]. (C and D) *Eutintinnus elongatus*. (E and F) *Dadayiella ganymedes*. (G) *Helicostomella subulata*. (H and I) *Cyttarocylis* sp. (J and K) *Rhabdonella spiralis*. I, II, microtubular ribbons I, II; Kd, kinetodesmal fibrils; Pc, postciliary ribbons. Scale bars = 250 nm (A, F, H, J, K), 500 nm (C, D, G, I), and 1 µm (B and E)

**FIGURE 2:**
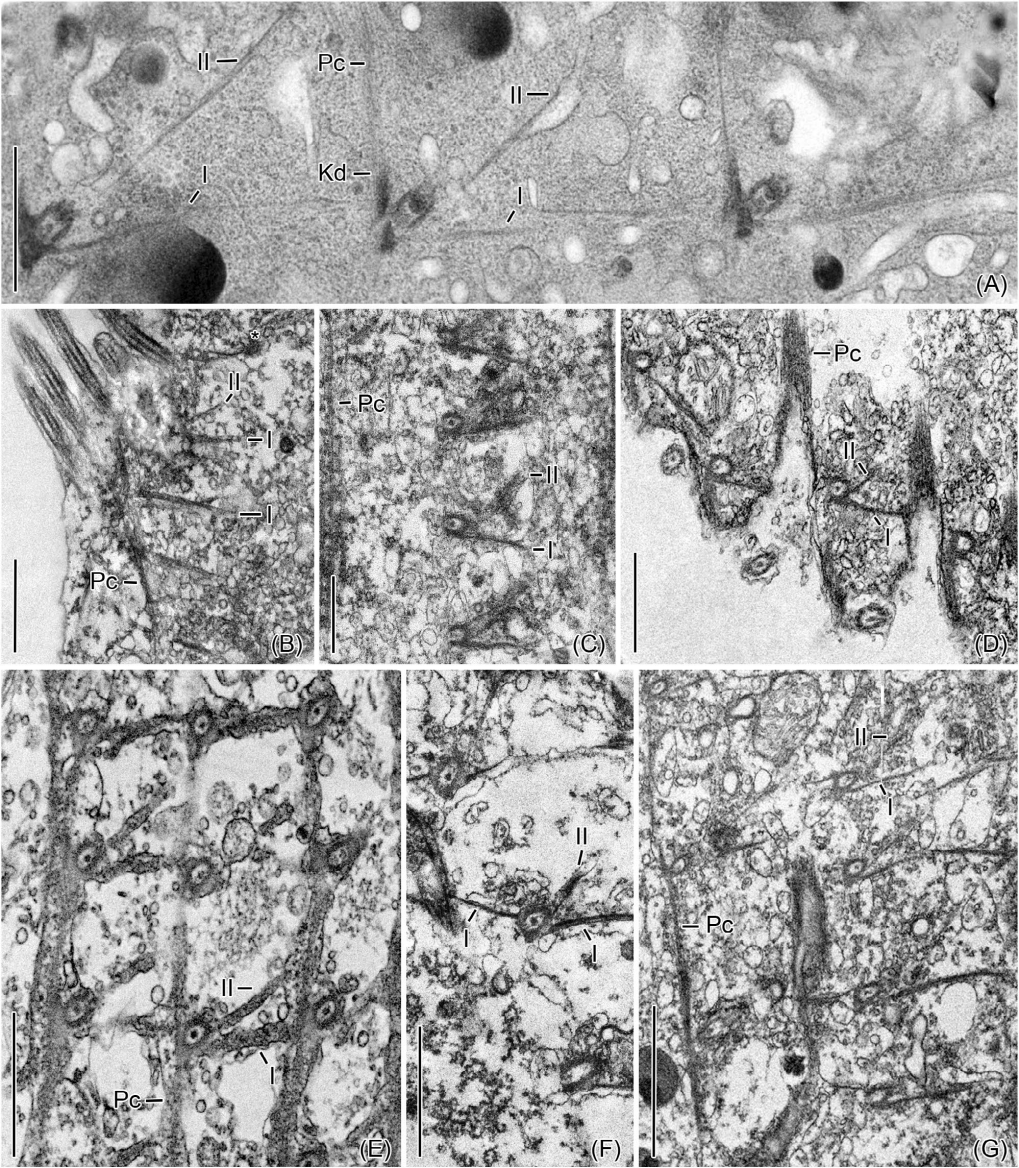
Transmission electron micrographs of tangential cell sections showing a network of kinetid‐associated fibrillar structures in the ciliated anterior portions of the tintinnids. (A) *Schmidingerella meunieri* [from Gruber et al. (2018)]. (B) *Amphorides minor*. A section through the posterior basal bodies of the obliquely orientated dikinetids and their fibrillar associates. The asterisk marks a basal body. (C) *Eutintinnus elongatus*. (D) *Dadayiella ganymedes*. (E) *Helicostomella subulata*. (F) *Cyttarocylis* sp. (G) *Rhabdonella spiralis*. I, II, microtubular ribbons I, II; Kd, kinetodesmal fibrils; Pc, postciliary ribbons. Scale bars = 1 µm (A–E, F) and 2 µm (G)

*Amphorides minor* (Jörgensen, 1924) Strand, 1928; Tintinnidae Claparède and Lachmann, 1858 (Table 2; Figures 1D,E and 3B). The somatic ciliature is exclusively composed of dikinetids which are clockwise inclined, forming angles of 17° ± 11° (2°–33°, *n* = 8) to the kinety axes. The structures associated with the dikinetids are identical to those in *Schmidingerella* and form a similar network.

*Eutintinnus elongatus* (Jörgensen, 1924) Kofoid and Campbell, 1939; Eutintinnidae Bachy et al., 2012 (Table 2; Figures 1F, 2C,D, and 3C). The dikinetids are only slightly clockwise inclined, forming angles of 5° ± 3° (2°–9°, *n* = 6) to the kinety axes. The structures associated with the dikinetids and monokinetids as well as the networks are identical to those in *Schmidingerella*, except for the transverse ribbons, which could not be detected.

*Dadayiella ganymedes* (Entz, 1884) Kofoid and Campbell, 1929; incertae sedis in Xystonellidae Kofoid and Campbell, 1929 (Table 2; Figures 1G,H, 2E,F, and 3D). The dikinetids are only slightly clockwise inclined, forming angles of 6° ± 2° (3°–10°, *n* = 7) to the kinety axes. The structures associated with the dikinetids and monokinetids as well as the networks are identical to those in *Schmidingerella*, except for the transverse ribbons, which could not be detected.

*Helicostomella subulata* (Ehrenberg, 1834) Jörgensen, 1924; incertae sedis in Tintinnina Kofoid and Campbell, 1929 (Table 2; Figures 1I,J, 2G, and 3E). The dikinetids are clockwise inclined, forming angles of about 8° ± 4° (2°–14°, *n* = 6) to the kinety axes. The structures associated with the dikinetids and monokinetids as well as the networks are identical to those in *Schmidingerella*; only the transverse ribbons are not clearly visible (Figure 1I).

*Cyttarocylis* sp.; Cyttarocylididae Kofoid and Campbell, 1929 (Table 2; Figures 1K,L, 2H,I, and 3F). The inclination of the dikinetids could not be measured. The structures associated with the dikinetids and monokinetids as well as the networks are identical to those in *Schmidingerella*, except for the transverse ribbons, which could not be detected.

*Rhabdonella spiralis* (Fol, 1881) Brandt, 1906; Rhabdonellidae Kofoid and Campbell, 1929 (Table 2; Figures 1M,N, 2J,K, and 3G). The dikinetids are clockwise inclined, forming angles of about 14° ± 3° (10°–18°, *n* = 4) to the kinety axes. The structures associated with the dikinetids and monokinetids as well as the networks are identical to those in *Schmidingerella*.

Parasomal sacs could be found neither in the cryofixed specimens of *Schmidingerella meunieri* nor in any of the chemically fixed tintinnid species.

## DISCUSSION

### Taxon coverage

Fourteen families of extant tintinnids have been compiled by Santoferrara and McManus (2021). Five of them plus one representative of the molecular tintinnid clade 11 were analysed in the present paper regarding the ultrastructure of their somatic kinetids (Figure S2). *Amphorides* is representative of the Tintinnidae, *Cyttarocylis* of the Cyttarocylididae, *Eutintinnus* of the Eutintinnidae, and *Schmidingerella* and *Rhabdonella* of the Rhabdonellidae; *Dadayiella* is assigned incertae sedis to the Xystonellidae, and *Helicostomella* is placed in the molecular tintinnid clade 11, and thus affiliated incertae sedis with the Tintinnina. Hedin (1976) provided some information on the monokinetids of *Ptychocylis minor* representing a further family, namely the Ptychocylididae Kofoid and Campbell, 1929. The previously mentioned genera and families possess hard loricae and are scattered across the molecular phylogeny (Santoferrara et al., 2017; Zhang et al., 2017), but do not cover the basally branching Tintinnidiidae with their soft loricae.

In the tintinnid genera studied here, the somatic ciliature is composed of some dikinetids and many monokinetids, except for *Amphorides* with exclusively dikinetidal somatic ciliary rows and without specialised kineties and ciliary fields (Figure 1D,E; Bai et al., 2020). The anteriormost dikinetids of its kineties have two cilia, while the following kinetids have cilia associated only with the posterior basal bodies.

### Comparison of tintinnids

**FIGURE 4:**
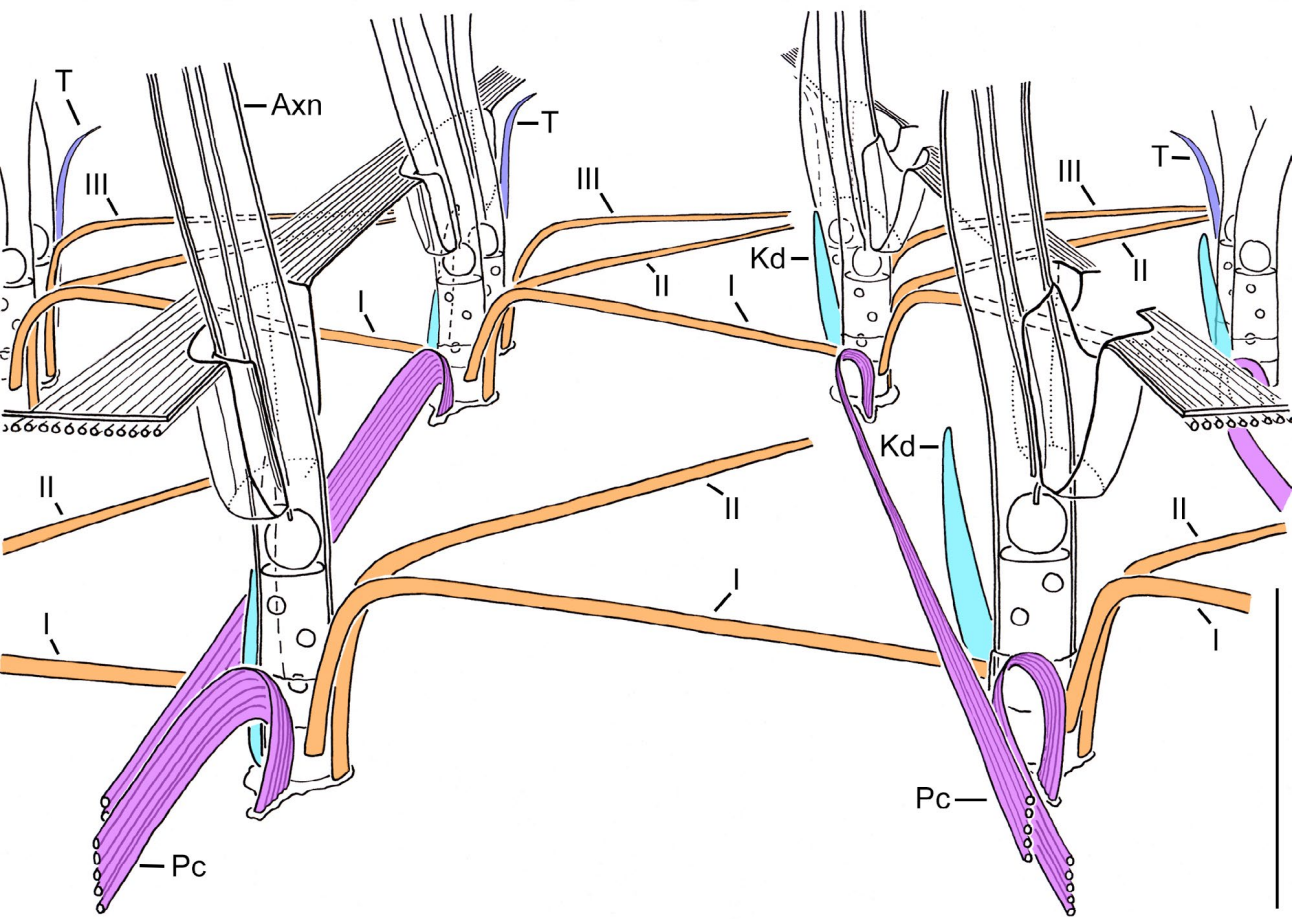
Scheme of a right or left ciliary field with a dikinetid at the anterior ends of the otherwise monokinetidal ciliary rows. It summarises the observations on kinetid‐associated structures in the tintinnids analysed in the present study and from the previous investigation by Gruber et al. (2018); please, note that *Amphorides* has exclusively dikinetidal ciliary rows and that transverse ribbons could not be detected in all tintinnids. In all taxa, the extraordinary ribbons I–III (orange) form together with the overlapping postciliary ribbons (purple) a network in the ciliated cell portion. The monokinetids correspond to the posterior dikinetidal basal bodies regarding the associated structures. The cilia insert in ciliary pits, and longitudinally orientated microtubules form a layer underneath the cell membrane. I–III, extraordinary ribbons I–III; Axn, axoneme; Kd, kinetodesmal fibrils; Pc, postciliary ribbons; T, transverse ribbons. Scale bar = 1 µm

The comparison of the somatic infraciliature in the seven tintinnid genera analysed here revealed consistently the three extraordinary ribbons I–III in dikinetids (Figure 1A–N), the two extraordinary ribbons I and II in monokinetids (Figure 2A–K), and a network in the anterior cell portion formed by these ribbons together with the overlapping postciliary ribbons (Figures 3A–G and 4). The reinvestigation of Hedin’s (1976, figures 8 and 10) micrographs of *Ptychocylis minor* by Gruber et al. (2018) discovered identical structures associated with the monokinetids, namely the ribbons I and II plus overlapping postciliary ribbons; very likely, they also form a network in the ciliated anterior cell portion. Despite the congruent occurrence of the three ribbons and the overlapping postciliary ribbons, a considerable variability emerged concerning the attachment sites of the ribbons I–III at the basal bodies, their curvatures, angles, and widths (numbers of the constituting microtubules). This variability might be attributed to different section planes or intrinsic deviations; further studies are thus required for differentiating these two factors. Likewise, they have to verify the general presence of transverse ribbons in tintinnid dikinetids, as they were only clearly visible in *Amphorides*,*Schmidingerella*, and *Rhabdonella*, while their presence was somewhat uncertain in *Helicostomella*, and they were not visible in *Eutintinnus*,*Dadayiella*, and *Cyttarocylis*. The fact that the monokinetids have the same structure than the posterior dikinetidal basal bodies further strengthens the kinetid transformation hypothesis established by Agatha and Strüder‐Kypke (2014). The overlapping postciliary ribbons of tintinnids differ from the ‘true’ postciliodesmata in the Postciliodesmatophora as defined by Lynn (2008, 2017) due to the absence of microtubules linking the particular ribbons.

The most parsimonious assumption inferred from the observed distribution of this type of somatic kinetids and the associated microtubular network among tintinnids is an evolution of these structures in at least the sistergroup of the Tintinnidiidae (Figure S2). The latter are widely restricted to freshwater and (frequently brackish) coastal waters and comprise three genera: *Tintinnidium* Saville‐Kent, 1881; *Membranicola* Foissner, Berger and Schaumburg, 1999; and *Antetintinnidium* Ganser and Agatha, 2019. Protargol‐stained *Antetintinnidium mucicola* specimens display an uncommon arrangement of argyrophilic fibres (Ganser & Agatha, 2019). While the kinetids of a kinety seem to be linked by subjacent postciliary ribbons as in other protargol‐stained tintinnids, a unique fibre each extends in some distance parallel to the kineties’ left sides. Some of its kinetids show associated fibrillar structures, which extend horizontally leftwards and apparently abut on the longitudinal fibres. Since these findings from protargol‐stained cells cannot be reconciled with the kinetid structures described above, transmission electron microscopic data on this basally branching family are required.

The microtubular network enhances the rigidity of the tintinnid's ciliated anterior cell portion. If it will also be verified in the Tintinnidiidae, its development might be connected with the acquisition of the lorica in tintinnids and/or with the high motility of the somatic cilia being potentially involved in lorica construction and waste removal (vs. the cilia are less mobile in aloricate choreotrichids and oligotrichids). Independent of the network's supposed function, the numerous and well‐developed kinetid‐associated structures contradict Raikov et al. (1975) and Lynn (1981) who hypothesised that when one associated structure (kinetodesmal fibril, transverse ribbon or postciliary ribbon) is well developed, the adjacent ones are reduced. On the contrary, tintinnids not only have all common kinetid‐associated structures, including strong overlapping postciliary ribbons, but also three further conspicuous ribbons (ribbons I–III).

### Somatic kinetids in other spirotrichs

The tintinnids form a morphologically and genetically supported monophylum, whereas the related aloricate choreotrichids are not monophyletic. Ultrastructural data are only available for the highly aberrant monokinetids with a double‐rowed ‘transverse’ ribbon in the aloricate choreotrichid family Strobilidiidae (Grim, 1987) and can thus not be extrapolated to the other families (Table 1). Protargol‐stained specimens of the strombidinopsid genera *Strombidinopsis* and *Parastrombidium* display argyrophilic structures extending on the right of the dikinetidal somatic kineties (Lynn et al., 1991; Xu et al., 2007) probably representing overlapping postciliary ribbons. In the oligotrichid *Limnostrombidium viride*, Bardele et al. (2018) observed an arrangement of electron‐dense material adjacent to the dikinetids resembling the attachment sites of the ribbons I–III. While further ultrastructural studies are thus needed to elucidate the distribution of the overlapping postciliary ribbons and the extraordinary ribbons I–III among aloricate choreotrichids, overlapping postciliary ribbons and a microtubular network similar to that described for the tintinnids are highly likely absent in oligotrichids (Bardele et al., 2018; Fauré‐Fremiet & Ganier, 1970; Modeo et al., 2003). Likewise, the ribbons I–III and overlapping postciliary ribbons have not been reported from any other spirotrich (Table 1). Hence, we agree with Lynn (2008) in that the structural conservatism hypothesis does not hold for the spirotrich ciliates and conclude that the ultrastructural data are currently too scarce regarding the Oligotrichea (Table 1) to characterise its orders (Oligotrichida, Lynnellida, and Choreotrichida) or suborders/families by particular types of somatic kinetids.

## Supporting information

Fig S1‐S2
