# Supplementary material for: Somatic infraciliature in tintinnid ciliates (Alveolata, Ciliophora, Spirotricha): An ultrastructural comparison*
Source: J Eukaryot Microbiol. 2022 Jan 30;69(2):e12885. doi: 10.1111/jeu.12885 (PMC9306737; doi:10.1111/jeu.12885)
Supplement: Supplementary file 1 — Fig S1‐S2 [file JEU-69-0-s001.pdf]

SUPPORTING INFORMATION

Somatic Infraciliature in Tintinnid Ciliates (Alveolata, Ciliophora, Spirotricha): An Ultrastructural Comparison

Sabine Agatha[a], Michael S. Gruber[b], Heidi Bartel[a], Birgit Weißenbacher[a]

[a] Department of Biosciences, Paris Lodron University of Salzburg, 5020 Salzburg, Austria
[b] Hieronymus-Illustrations, Zehentmaiergasse 10/17, Salzburg, Austria

Correspondence
S. Agatha, Department of Biosciences, Paris Lodron University of Salzburg, Hellbrunnerstrasse 34, 5020 Salzburg, Austria
E-mail: sabine.agatha@plus.ac.at

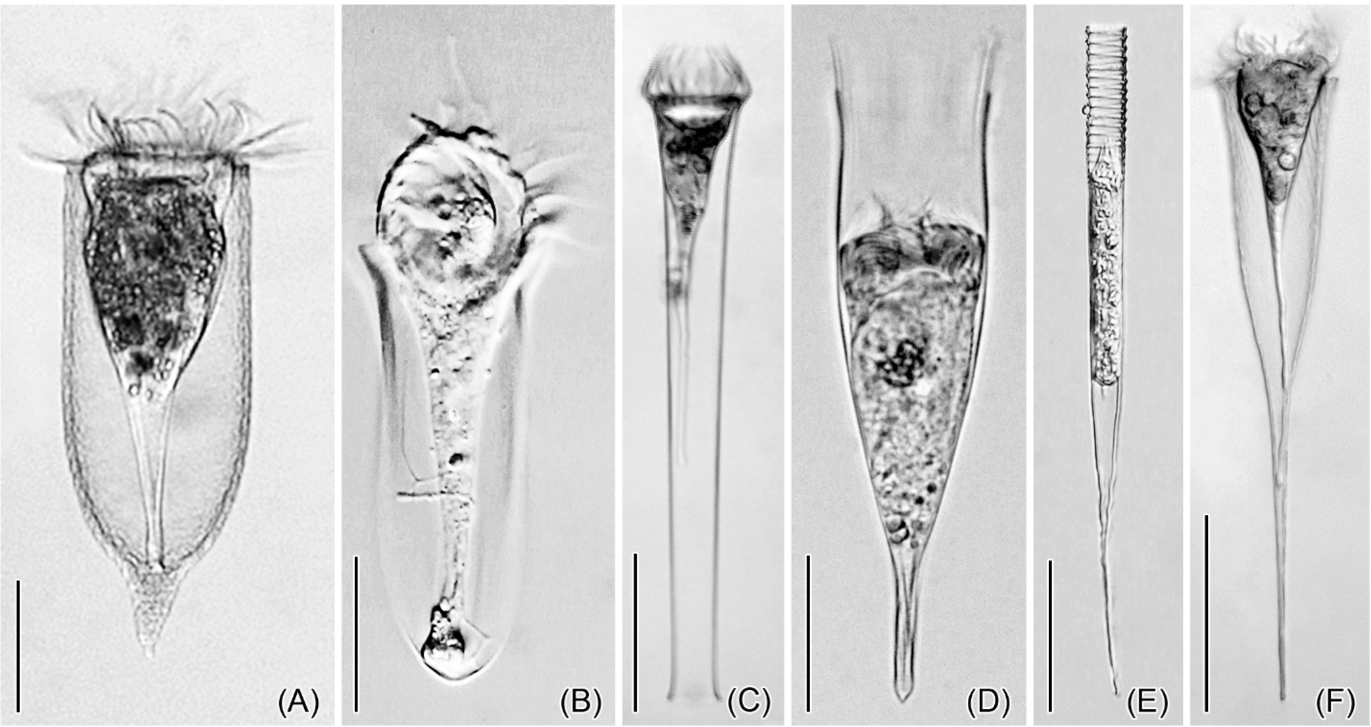

FIGURE S1 Light micrographs of the tintinnid taxa ultrastructurally investigated in the present study, except for *Cyttarocylis* sp. (A) *Schmidingerella meunieri*, Rhabdonellidae. (B) *Amphorides minor*, Tintinnidae. (C) *Eutintinnus elongatus*, Eutintinnidae. (D) *Dadayiella ganymedes*, incertae sedis in Xystonellidae. (E) *Helicostomella subulata*, incertae sedis in Tintinnina. (F) *Rhabdonella spiralis*, Rhabdonellidae. Scale bars = 50 µm (A), 30 µm (B and D), 100 µm (C, E, F).

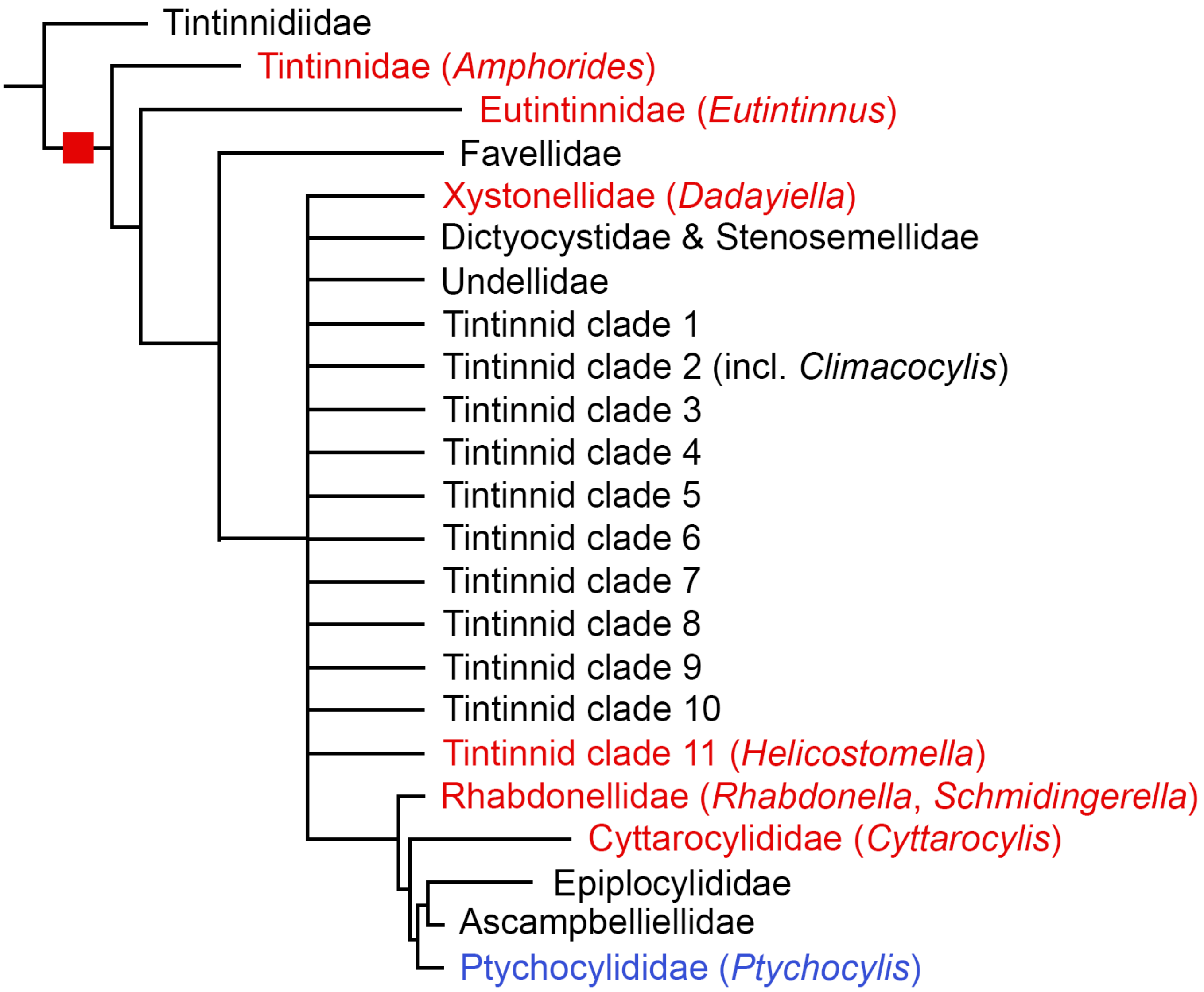

FIGURE S2 Simplified consensus tintinnid phylogeny inferred from 18S rDNA gene sequences (modified from Santoferrara and McManus, 2020). The genera and families ultrastructurally investigated in the present study are marked by red. The first occurrence of the extraordinary ribbons I-III and the microtubular networks inferred from our findings is denoted by a red square. Gruber et al. (2018) reinvestigated micrographs of monokinetids in *Ptychocylis minor*, family Ptychocylididae (marked by blue), published by Hedin (1976) and discovered overlapping postciliary ribbons and the extraordinary ribbons I and II.
